# Properties of Zinc Oxide Nanoparticles and Their Activity Against Microbes

**DOI:** 10.1186/s11671-018-2532-3

**Published:** 2018-05-08

**Authors:** Khwaja Salahuddin Siddiqi, Aziz ur Rahman, Tajuddin, Azamal Husen

**Affiliations:** 1 Department of Chemistry, Aligarh Muslim University, Aligarh, Uttar Pradesh 202002 India; 2 Department of Saidla (Unani Pharmacy), Aligarh Muslim University, Aligarh, Uttar Pradesh 202002 India; 3 Department of Biology, College of Natural and Computational Sciences, University of Gondar, P.O. Box #196, Gondar, Ethiopia

**Keywords:** Zinc oxide nanoparticles, Microorganisms, Antimicrobial, Toxicity, Mechanism, Biodistribution

## Abstract

Zinc oxide is an essential ingredient of many enzymes, sun screens, and ointments for pain and itch relief. Its microcrystals are very efficient light absorbers in the UVA and UVB region of spectra due to wide bandgap. Impact of zinc oxide on biological functions depends on its morphology, particle size, exposure time, concentration, pH, and biocompatibility. They are more effective against microorganisms such as *Bacillus subtilis*, *Bacillus megaterium*, *Staphylococcus aureus*, *Sarcina lutea*, *Escherichia coli*, *Pseudomonas aeruginosa*, *Klebsiella pneumonia*, *Pseudomonas vulgaris*, *Candida albicans*, and *Aspergillus niger*. Mechanism of action has been ascribed to the activation of zinc oxide nanoparticles by light, which penetrate the bacterial cell wall via diffusion. It has been confirmed from SEM and TEM images of the bacterial cells that zinc oxide nanoparticles disintegrate the cell membrane and accumulate in the cytoplasm where they interact with biomolecules causing cell apoptosis leading to cell death.

## Background

Nanotechnology deals with the manufacture and application of materials with size of up to 100 nm. They are widely used in a number of processes that include material science, agriculture, food industry, cosmetic, medical, and diagnostic applications [1–10]. Nanosize inorganic compounds have shown remarkable antibacterial activity at very low concentration due to their high surface area to volume ratio and unique chemical and physical features [11]. In addition, these particles are also more stable at high temperature and pressure [12]. Some of them are recognized as nontoxic and even contain mineral elements which are vital for human body [13]. It has been reported that the most antibacterial inorganic materials are metallic nanoparticles and metal oxide nanoparticles such as silver, gold, copper, titanium oxide, and zinc oxide [14, 15].

Zinc is an essential trace element for human system without which many enzymes such as carbonic anhydrase, carboxypeptidase, and alcohol dehydrogenase become inactive, while the other two members, cadmium and mercury belonging to the same group of elements having the same electronic configuration, are toxic. It is essential for eukaryotes because it modulates many physiological functions [16, 17]. Bamboo salt, containing zinc, is used as herbal medicine for the treatment of inflammation by regulating caspase-1 activity. Zinc oxide nanoparticles have been shown to reduce mRNA expression of inflammatory cytokines by inhibiting the activation of NF-kB (nuclear factor kappa B cells) [18].

Globally, bacterial infections are recognized as serious health issue. New bacterial mutation, antibiotic resistance, outbreaks of pathogenic strains, etc. are increasing, and thus, development of more efficient antibacterial agents is demand of the time. Zinc oxide is known for its antibacterial properties from the time immemorial [19]. It had been in use during the regime of Pharaohs, and historical records show that zinc oxide was used in many ointments for the treatment of injuries and boils even in 2000 BC [20]. It is still used in sun screen lotion, as a supplement, photoconductive material, LED, transparent transistors, solar cells, memory devices [21, 22], cosmetics [23, 24], and catalysis [25]. Although considerable amount of ZnO is produced every year, very small quantity is used as medicine [26]. The US Food and Drug Administration has recognized (21 CFR 182.8991) zinc oxide as safe [27]. It is characterized by photocatalytic and photooxidizing properties against biochemicals [28].

Zinc oxide has been classified by EU hazard classification as N; R50-53 (ecotoxic). Compounds of zinc are ecotoxic for mammals and plants in traces [29, 30]. Human body contains about 2–3 g of zinc, and the daily requirement is 10–15 mg [29, 31]. No report has demonstrated carcinogenicity, genotoxicity, and reproduction toxicity in humans [29, 32]. However, zinc powder inhaled or ingested may produce a condition called zinc fever, which is followed by chill, fever, cough, etc.

Morphology of zinc oxide nanoparticles depends on the process of synthesis. They may be nanorods, nanoplates [33–35], nanospheres [36], nanoboxes [35], hexagonal, tripods [37], tetrapods [38], nanowires, nanotubes, nanorings [39–41], nanocages, and nanoflowers [42, 43]. Zinc oxide nanoparticles are more active against gram-positive bacteria relative to other NPs of the same group of elements. Ready to eat food is more prone to infection by *Salmonella*, *Staphylococcus aureus*, and *E. coli* which pose a great challenge to food safety and quality. The antimicrobial compounds are incorporated in the packed food to prevent them from damage. Antimicrobial packaging contains a nontoxic material which inhibits or slows down the growth of microbes present in food or packaging material [44]. An antimicrobial substance for human consumption must possess the following properties.

- It should be nontoxic.
- It should not react with food or container.
- It should be of good taste or tasteless.
- It should not have disagreeable smell.

Zinc oxide nanoparticle is one such inorganic metal oxide which fulfills all the above requirements, and hence, it can safely be used as medicine, preservative in packaging, and an antimicrobial agent [45, 46]. It easily diffuses into the food material, kill the microbes, and prevent human being from falling ill. In accordance with the regulations 1935/2004/EC and 450/2009/EC of the European Union, active packaging is defined as active material in contact with food with ability to change the composition of the food or the atmosphere around it [47]. Therefore, it is commonly used as preservative and incorporated in polymeric packaging material to prevent food material from damage by microbes [48]. Zinc oxide nanoparticles have been used as an antibacterial substance against *Salmonella typhi* and *S. aureus* in vitro. Of all the metal oxide nanoparticles studied thus far, zinc oxide nanoparticles exhibited the highest toxicity against microorganisms [49]. It has also been demonstrated from SEM and TEM images that zinc oxide nanoparticles first damage the bacterial cell wall, then penetrate, and finally accumulate in the cell membrane. They interfere with metabolic functions of the microbes causing their death. All the characteristics of the zinc oxide nanoparticles depend on their particle size, shape, concentration, and exposure time to the bacterial cell. Further, biodistribution studies of zinc oxide nanoparticles have also been examined. For instance, Wang et al. [50] have investigated the effect of long-term exposure of zinc oxide nanoparticle on biodistribution and zinc metabolism in mice over 3 to 35 weeks. Their results showed minimum toxicity to mice when they were exposed to 50 and 500 mg/kg zinc oxide nanoparticle in diet. At higher dose of 5000 mg/kg, zinc oxide nanoparticle decreased body weight but increased the weight of the pancreas, brain, and lung. Also, it increased the serum glutamic-pyruvic transaminase activity and mRNA expression of zinc metabolism-related genes such as metallothionein. Biodistribution studies showed the accumulation of sufficient quantity of zinc in the liver, pancreas, kidney, and bones. Absorption and distribution of zinc oxide nanoparticle/zinc oxide microparticles are largely dependent on the particle size. Li et al. [51] have studied biodistribution of zinc oxide nanoparticles fed orally or through intraperitoneal injection to 6 weeks old mice. No obvious adverse effect was detected in zinc oxide nanoparticles orally treated mice in 14 days study. However, intraperitoneal injection of 2.5 g/kg body weight given to mice showed accumulation of zinc in the heart, liver, spleen, lung, kidney, and testes. Nearly ninefold increase in zinc oxide nanoparticle in the liver was observed after 72 h. Zinc oxide nanoparticles have been shown to have better efficiency in liver, spleen, and kidney biodistribution than in orally fed mice. Since zinc oxide nanoparticles are innocuous in low concentrations, they stimulate certain enzymes in man and plants and suppress diseases. Singh et al. [52] have also been recently reviewed the biosynthesis of zinc oxide nanoparticle, their uptake, translocation, and biotransformation in plant system.

In this review, we have attempted to consolidate all the information regarding zinc oxide nanoparticles as antibacterial agent. The mechanism of interaction of zinc oxide nanoparticles against a variety of microbes has also been discussed in detail.

## Antimicrobial Activity of Zinc Oxide Nanoparticles

It is universally known that zinc oxide nanoparticles are antibacterial and inhibit the growth of microorganisms by permeating into the cell membrane. The oxidative stress damages lipids, carbohydrates, proteins, and DNA [53]. Lipid peroxidation is obviously the most crucial that leads to alteration in cell membrane which eventually disrupt vital cellular functions [54]. It has been supported by oxidative stress mechanism involving zinc oxide nanoparticle in *Escherichia coli* [55]. However, for bulk zinc oxide suspension, external generation of H_2_O_2_ has been suggested to describe the anti-bacterial properties [56]. Also, the toxicity of nanoparticles, releasing toxic ions, has been considered. Since zinc oxide is amphoteric in nature, it reacts with both acids and alkalis giving Zn^2+^ ions.

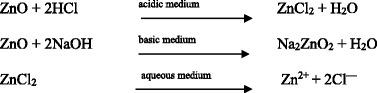



The free Zn^2+^ ions immediately bind with the biomolecules such as proteins and carbohydrates, and all vital functions of bacteria cease to continue. The toxicity of zinc oxide, zinc nanoparticles, and ZnSO_4_·7H_2_O has been tested (Table 1) against *Vibrio fischeri*. It was found that ZnSO_4_·7H_2_O is six times more toxic than zinc oxide nanoparticles and zinc oxide. The nanoparticles are actually dispersed in the solvent, not dissolved, and therefore, they cannot release Zn^2+^ ions. The bioavailability of Zn^2+^ ions is not always 100% and may invariably change with physiological pH, redox potential, and the anions associated with it such as Cl^−^ or SO_4_^2−^.

**Table 1:** The toxicity (30-min EC_50_, EC_20_ and NOEC, and MIC) of metal oxide aqueous suspensions CuSO_4_ and ZnSO_4_·7H_2_O to bacteria *Vibrio fischeri* [59]

Chemical	Toxicity to *Vibrio fischeri*, EC_50_, EC_20_, NOEC, and MIC (mg l^− 1^)			
	EC_50_ ± SD	EC_20_ ± SD	NOEC	MIC
ZnO	1.8 ± 0.1 (1.4 ± 0.08)	1.0 ± 0.4 (0.8 ± 0.3)	1.0 (0.8)	200 (160)
Nano-ZnO	1.9 ± 0.2 (1.5 ± 0.16)	0.9 ± 0.4 (0.7 ± 0.3)	0.75 (0.6)	100 (80)
ZnSO_4_·7H_2_O	1.1 ± 0.25 (0.25 ± 0.06)	0.8 ± 0.3 (0.2 ± 0.1)	0.5 (0.11)	10 (2.0)
CuO	3811 ± 1012 (3049 ± 819)	903 ± 457 (722 ± 366)	313 (250)	20,000 (16,000)
Nano-CuO	79 ± 27 (63 ± 22)	24 ± 5 (19 ± 4)	16 (12)	200 (160)
CuSO_4_	1.6 ± 0.29 (0.64 ± 0.12)	0.9 ± 0.3 (0.36 ± 0.12)	0.63 (0.25)	2.5 (1.0)
TiO_2_	> 20,000	> 20,000	> 20,000	> 20,000
Nano-TiO_2_	> 20,000	> 20,000	> 20,000	> 20,000

Solubility of zinc oxide (1.6–5.0 mg/L) in aqueous medium is higher than that of zinc oxide nanoparticles (0.3–3.6 mg/L) in the same medium [57] which is toxic to algae and crustaceans. Both nano-zinc oxide and bulk zinc oxide are 40–80-fold less toxic than ZnSO_4_ against *V. fischeri*. The higher antibacterial activity of ZnSO_4_ is directly proportional to its solubility releasing Zn^2+^ ions, which has higher mobility and greater affinity [58] toward biomolecules in the bacterial cell due to positive charge on the Zn^2+^ and negative charge on the biomolecules.

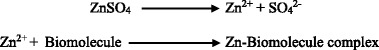



Since zinc oxide and its nanoparticles have limited solubility, they are less toxic to the microbes than highly soluble ZnSO_4_·7H_2_O. However, it is not essential for metal oxide nanoparticles to enter the bacterial cell to cause toxicity [59]. Contact between nanoparticles and the cell wall is sufficient to cause toxicity. If it is correct, then large amounts of metal nanoparticles are required so that the bacterial cells are completely enveloped and shielded from its environment leaving no chance for nutrition to be absorbed to continue life process. Since nanoparticles and metal ions are smaller than the bacterial cells, it is more likely that they disrupt the cell membrane and inhibit their growth.

A number of nanosized metal oxides such as ZnO, CuO, Al_2_O_3_, La_2_O_3_, Fe_2_O_3_, SnO_2_, and TiO_2_ have been shown to exhibit the highest toxicity against *E. coli* [49]. Zinc oxide nanoparticles are externally used for the treatment of mild bacterial infections, but the zinc ion is an essential trace element for some viruses and human beings which increase enzymatic activity of viral integrase [45, 60, 61]. It has also been supported by an increase in the infectious pancreatic necrosis virus by 69.6% when treated with 10 mg/L of Zn [46]. It may be due to greater solubility of Zn ions relative to ZnO alone. The SEM and TEM images have shown that zinc oxide nanoparticles damage the bacterial cell wall [55, 62] and increase permeability followed by their accumulation in *E. coli* preventing their multiplication [63].

In the recent past, antibacterial activity of zinc oxide nanoparticle has been investigated against four known gram-positive and gram-negative bacteria, namely *Staphylococcus aureus*, *E. coli*, *Salmonella typhimurium*, and *Klebsiella pneumoniae*. It was observed that the growth-inhibiting dose of the zinc oxide nanoparticles was 15 μg/ml, although in the case of *K. pneumoniae*, it was as low as 5 μg/ml [63, 64]. It has been noticed that with increasing concentration of nanoparticles, growth inhibition of microbes increases. When they were incubated over a period of 4–5 h with a maximum concentration of zinc oxide nanoparticles of 45 μg/ml, the growth was strongly inhibited. It is expected that if the incubation time is increased, the growth inhibition would also increase without much alteration in the mechanism of action [63].

It has been reported that the metal oxide nanoparticles first damage the bacterial cell membrane and then permeate into it [64]. It has also been proposed that the release of H_2_O_2_ may be an alternative to anti-bacterial activity [65]. This proposal, however, requires experimental proof because the mere presence of zinc oxide nanoparticle is not enough to produce H_2_O_2_. Zinc nanoparticles or zinc oxide nanoparticles of extremely low concentration cannot cause toxicity in human system. Daily intake of zinc via food is needed to carry out the regular metabolic functions. Zinc oxide is known to protect the stomach and intestinal tract from damage by *E. coli *[65]. The pH in the stomach varies between 2 to 5, and hence, zinc oxide in the stomach can react with acid to produce Zn^2+^ ions. They can help in activating the enzyme carboxy peptidase, carbonic anhydrase, and alcohol dehydrogenase which help in the digestion of carbohydrate and alcohol. Premanathan et al. [66] have reported the toxicity of zinc oxide nanoparticles against prokaryotic and eukaryotic cells. The MIC of zinc oxide nanoparticles against *E. coli*, *Pseudomonas aeruginosa*, and *S. aureus* were found to be 500 and 125 μg/ml, respectively. Two mechanisms of action have been proposed for the toxicity of zinc oxide nanoparticles, namely (1) generation of ROS and (2) induction of apoptosis. Metal oxide nanoparticles induce ROS production and put the cells under oxidative stress causing damage to cellular components, i.e., lipids, proteins, and DNA [67–69]. Zinc oxide nanoparticles, therefore, induce toxicity through apoptosis. They are relatively more toxic to cancer cells than normal cells, although they cannot distinguish between them.

Recently, Pati et al. [70] have shown that zinc oxide nanoparticles disrupt bacterial cell membrane integrity, reduce cell surface hydrophobicity, and downregulate the transcription of oxidative stress-resistance genes in bacteria. They enhance intracellular bacterial killing by inducing ROS production. These nanoparticles disrupt biofilm formation and inhibit hemolysis by hemolysin toxin produced by pathogens. Intradermal administration of zinc oxide nanoparticles was found to significantly reduce the skin infection and inflammation in mice and also improved infected skin architecture.

### Solubility and Concentration-Dependent Activity of Zinc Oxide Nanoparticle

Nanoparticles have also been used as a carrier to deliver therapeutic agents to treat bacterial infection [1, 9]. Since zinc oxide nanoparticles up to a concentration of 100 μg/ml are harmless to normal body cells, they can be used as an alternative to antibiotics. It was found that 90% bacterial colonies perished after exposing them to a dose of 500–1000 μg/ml of zinc oxide nanoparticles only for 6 h. Even the drug-resistant *S. aureus*, *Mycobacterium smegmatis*, and *Mycobacterium bovis* when treated with zinc oxide nanoparticles in combination with a low dose of anti-tuberculosis drug, rifampicin (0.7 μg/ml), a significant reduction in their growth was observed. These pathogens were completely destroyed when incubated for 24 h with 1000 μg/ml of zinc oxide nanoparticles. It is, therefore, concluded that if the same dose is repeated, the patient with such infective diseases may be completely cured. It was also noted that the size of zinc oxide nanoparticles ranging between 50 and 500 nm have identical effect on bacterial growth inhibition.

Cytotoxicity of zinc oxide has been studied by many researchers in a variety of microbes and plant systems [71–74]. Toxicity of zinc oxide nanoparticles is concentration and solubility dependent. It has been shown that maximum exposure concentration of zinc oxide (125 mg/l) suspension released 6.8 mg/l of Zn^2+^ ions. Toxicity is a combined effect of zinc oxide nanoparticles and Zn^2+^ ions released in the aqueous medium. However, minimal effect of metal ions was detected which suggests that the bacterial growth inhibition is mainly due to interaction of zinc oxide nanoparticles with microorganisms. The cytotoxic effect of a particular metal oxide nanoparticle is species sensitive which is reflected by the growth inhibition zone for several bacteria [75].

It has been suggested that growth inhibition of bacterial cells occurs mainly by Zn^2+^ ions which are produced by extracellular dissolution of zinc oxide nanoparticles [76]. Cho et al. [77] have concluded from their studies on rats that zinc oxide nanoparticles remain intact at around neutral or biological pH but rapidly dissolve under acidic conditions (pH 4.5) in the lysosome of the microbes leading to their death. This is true because in acidic condition, zinc oxide dissolves and Zn^2+^ ions are produced, which bind to the biomolecules inside the bacterial cell inhibiting their growth.

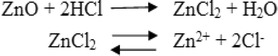



The zinc oxide nanoparticles have been shown to be cytotoxic to different primary immune-competent cells. The transcriptomics analysis showed that nanoparticles had a common gene signature with upregulation of metallothionein genes ascribed to the dissolution of the nanoparticles [78]. However, it could not be ascertained if the absorbed zinc was Zn^2+^ or zinc oxide or both, although smaller sized zinc oxide nanoparticles have greater concentration in the blood than larger ones (19 and > 100 nm). The efficiency of zinc oxide nanoparticles depends mainly on the medium of reaction to form Zn^2+^ and their penetration into the cell.

Chiang et al. [79] have reported that dissociation of zinc oxide nanoparticles results in destruction of cellular Zn homeostasis. The characteristic properties of nanoparticles and their impact on biological functions are entirely different from those of the bulk material [80]. Aggregation of nanoparticles influences cytotoxicity of macrophages, and their concentration helps in modulation of nanoparticle aggregation. Low concentration of zinc oxide nanoparticles is ineffective, but at higher concentration (100 μg/ml), they exhibited cytotoxicity which varies from one pathogen to another.

The inadvertent use of zinc oxide nanoparticles may sometime adversely affect the living system. Their apoptosis and genotoxic potential in human liver cells and cellular toxicity has been studied. It was found that a decrease in liver cell viability occurs when they are exposed to 14–20 μg/ml of zinc oxide nanoparticles for 12 h. It also induced DNA damage by oxidative stress. Sawai et al. [56] have demonstrated that ROS generation is directly proportional to the concentration of zinc oxide powder. ROS triggered a decrease in mitochondria membrane potential leading to apoptosis [81]. Cellular uptake of nanoparticles is not mandatory for cytotoxicity to occur.

### Size-Dependent Antibacterial Activity of Zinc Oxide Nanoparticles

**Fig. 1:**
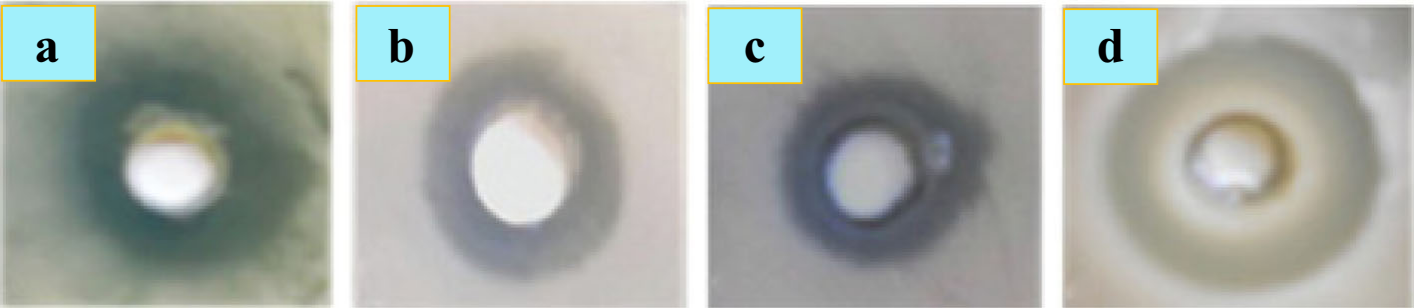
Antibacterial activity and/or zone of inhibition produced by zinc oxide nanoparticles against gram-positive and gram-negative bacterial strains namely **a** *Escherichia coli*, **b** *Staphylococcus aureus*, **c** *Pseudomonas aeruginosa*, and **d** *Bacillus subtilis* [82]

In a study, Azam et al. [82] have reported that the antimicrobial activity against both gram-negative (*E. coli* and *P. aeruginosa*) and gram-positive (*S.* and *Bacillus subtilis*) bacteria increased with increase in surface-to-volume ratio due to a decrease in particle size of zinc oxide nanoparticles. Moreover, in this investigation, zinc oxide nanoparticles have shown maximum (25 mm) bacterial growth inhibition against *B. subtilis* (Fig. 1).

It has been reported that the smaller size of zinc oxide nanoparticles exhibits greater antibacterial activity than microscale particles [83]. For instance, Au^55^ nanoparticles of 1.4-nm size have been demonstrated to interact with the major grooves of DNA which accounts for its toxicity [84]. Although contradictory results have been reported, many workers showed positive effect of zinc oxide nanoparticles on bacterial cells. However, Brayner et al. [63] from TEM images have shown that zinc oxide nanoparticle of 10–14 nm were internalized (when exposed to microbes) and damaged the bacterial cell membrane. It is also essential that the zinc/zinc oxide nanoparticles must not be toxic to human being since they are toxic to T cells above 5 mM [85] and to neuroblastoma cells above 1.2 mM [86]. Nair et al. [87] have exclusively explored the size effect of zinc oxide nanoparticles on bacterial and human cell toxicity. They have studied the influence of zinc oxide nanoparticles on both gram-positive and gram-negative bacteria and osteoblast cancer cell lines (MG-63).

It is known that antibacterial activity of zinc oxide nanoparticle is inversely proportional to their size and directly proportional to their concentration [88]. It has also been noticed that it does not require UV light for activation; it functions under normal or even diffused sunlight. Cytotoxic activity perhaps involves both the production of ROS and accumulation of nanoparticles in the cytoplasm or on the outer cell membrane. However, the production of H_2_O_2_ and its involvement in the activation of nanoparticles cannot be ignored. Raghupathi et al. [88] have synthesized zinc oxide nanoparticles from different zinc salts and observed that nanoparticles obtained from Zn(NO_3_)_2_ were smallest in size (12 nm) and largest in surface area (90.4). Authors have shown that the growth inhibition of *S. aureus* at a concentration of 6 mM of zinc oxide nanoparticles is size dependent. It has also been indicated from the viable cell determination during the exposure of bacterial cells to zinc oxide nanoparticles that the number of cells recovered decreased significantly with decrease in size of zinc oxide nanoparticles. Jones et al. [89] have shown that zinc oxide nanoparticles of 8-nm diameter inhibited the growth of *S. aureus*, *E. coli*, and *B. subtilis.* Zinc oxide nanoparticles ranging between 12 and 307 nm were selected and confirmed the relationship between antibacterial activity and their size. Their toxicity to microbes has been ascribed to the formation of Zn^2+^ ions from zinc oxide when it is suspended in water and also to some extent to a slight change in pH. Since Zn^2+^ ions are scarcely released from zinc oxide nanoparticles, the antibacterial activity is mainly owing to smaller zinc oxide nanoparticles. When the size is 12 nm, it inhibits the growth of *S. aureus*, but when the size exceeds 100 nm, the inhibitory effect is minimal [89].

### Shape, Composition, and Cytotoxicity of Zinc Oxide Nanoparticles

Zinc oxide nanoparticles have shown cytotoxicity in concentration-dependent manner and type of cells exposed due to different sensitivity [90, 91]. Sahu et al. [90] have highlighted the difference of cytotoxicity between particle size and different sensitivity of cells toward the particles of the same composition. In another recent study, Ng et al. [91] examined the concentration-dependent cytotoxicity in human lung MRC5 cells. Authors have reported the uptake and internalization of zinc oxide nanoparticles into the human lung MRC5 cells by using TEM investigation. These particles were noticed in the cytoplasm of the cells in the form of electron dense clusters, which are further observed to be enclosed by vesicles, while zinc oxide nanoparticles were not found in untreated control cells. Papavlassopoulos et al. [92] have synthesized zinc oxide nanoparticle tetrapods by entirely a novel route known as “Flame transport synthesis approach”. Tetrapods have different morphology compared to the conventionally synthesized zinc oxide nanoparticles. Their interaction with mammalian fibroblast cells in vitro has indicated that their toxicity is significantly lower than those of the spherical zinc oxide nanoparticles. Tetrapods exhibited hexagonal wurtzite crystal structure with alternating Zn^2+^ and O^2−^ ions with three-dimensional geometry. They block the entry of viruses into living cells which is further enhanced by precisely illuminating them with UV radiation. Since zinc oxide tetrapods have oxygen vacancies in their structure, the *Herpes simplex* viruses are attached via heparan sulfate and denied entry into body cells. Thus, they prevent HSV-1 and HSV-2 infection in vitro. Zinc oxide tetrapods may therefore be used as prophylactic agent against these viral infections. The cytotoxicity of zinc oxide nanoparticles also depends on the proliferation rate of mammalian cells [66, 93]. The surface reactivity and toxicity may also be varied by controlling the oxygen vacancy in zinc oxide tetrapods. When they are exposed to UV light, the oxygen vacancy in tetrapods is readily increased. Alternatively, the oxygen vacancy can be decreased by heating them in oxygen-rich environment. Thus, it is the unique property of zinc oxide tetrapods that can be changed at will which consequently alter their antimicrobial efficiency.

Animal studies have indicated an increase in pulmonary inflammation, oxidative stress, etc. on respiratory exposure to nanoparticles [94]. Yang et al. [95] have investigated the cytotoxicity, genotoxicity, and oxidative stress of zinc oxide nanoparticles on primary mouse embryo fibroblast cells. It was observed that zinc oxide nanoparticles induced significantly greater cytotoxicity than that induced by carbon and SiO_2_ nanoparticles. It was further confirmed by measuring glutathione depletion, malondialdehyde production, superoxide dismutase inhibition, and ROS generation. The potential cytotoxic effects of different nanoparticles have been attributed to their shape.

### Polymer-Coated Nanoparticles

Many bacterial infections are transmitted by contact with door knobs, key boards, water taps, bath tubs, and telephones; therefore, it is essential to develop and coat such surfaces with inexpensive advanced antibacterial substances so that their growth is inhibited. It is important to use such concentrations of antibacterial substances that they may kill the pathogens but spare the human beings. It may happen only if they are coated with a biocompatible hydrophilic polymer of low cost. Schwartz et al. [96] have reported the preparation of a novel antimicrobial composite material hydrogel by mixing a biocompatible poly (*N*-isopropylacrylamide) with zinc oxide nanoparticles. The SEM image of the composite film showed uniform distribution of zinc oxide nanoparticles. It exhibited antibacterial activity against *E. coli* at a very low zinc oxide concentration (1.33 mM). Also, the coating was found to be nontoxic toward mammalian cell line (N1H/3T3) for a period of 1 week. Zinc oxide/hydrogel nanocomposite may safely be used as biomedical coating to prevent people from contracting bacterial infections.

Although zinc oxide nanoparticles are stable, they have been further stabilized by coating them with different polymers such as polyvinyl pyrolidone (PVP), polyvinyl alcohol **(**PVA**),** poly (α, γ, l-glutamic acid) (PGA), polyethylene glycol (PEG), chitosan, and dextran [97, 98]. The antibacterial activity of engineered zinc oxide nanoparticles was examined against gram-negative and gram-positive pathogens, namely *E. coli* and *S. aureus* and compared with commercial zinc oxide powder. The polymer-coated spherical zinc oxide nanoparticles showed maximum bacterial cell destruction compared to bulk zinc oxide powder [99]. Since nanoparticles coated with polymers are less toxic due to their low solubility and sustained release, their cytotoxicity can be controlled by coating them with a suitable polymer.

### Effect of Particle Size and Shape of Polymer-Coated Nanoparticles on Antibacterial Activity

*E. coli* and *S. aureus* exposed to different concentrations of poly ethylene glycol (PEG)-coated zinc oxide nanoparticles (1–7 mM) of varying size (401 nm–1.2 μm) showed that the antimicrobial activity increases with decreasing size and increasing concentration of nanoparticles. However, the effective concentration in all these cases was above 5 mM. There occurs a drastic change in cell morphology of *E. coli* surface which can be seen from the SEM images of bacteria before and after their exposure to zinc oxide nanoparticles [84]. It has been nicely demonstrated by Nair et al. [87] that PEG-capped zinc oxide particles and zinc oxide nanorods are toxic to human osteoblast cancer cells (MG-63) at concentration above 100 μM. The PEG starch-coated nanorods/nanoparticles do not damage the healthy cells.

### In Vivo and In Vitro Antimicrobial Activity for Wound Dressing

Of all natural and synthetic wound dressing materials, the chitosan hydrogel microporous bandages laced with zinc oxide nanoparticles developed by Kumar et al. [100] are highly effective in treating burns, wounds, and diabetic foot ulcers. The nanoparticles of approximately 70–120 nm are dispersed on the surface of the bandage. The degradation products of chitosan were identified as d-glucosamine and glycosamine glycan. They are nontoxic to the cells because they are already present in our body for the healing of injury. The wound generally contains *P. aeruginosa*, *S. intermedicus*, and *S. hyicus* which were also identified from the swab of mice wound and successfully treated with chitosan zinc oxide bandage in about 3 weeks [100].

### Effect of Doping on Toxicity of Zinc Oxide Nanoparticles

Doping of zinc oxide nanoparticles with iron reduces the toxicity. The concentration of Zn^2+^ and zinc oxide nanoparticles is also an important factor for toxicity. The concentration that reduced 50% viability in microbial cells exposed to nano- and microsize zinc oxide is very close to the concentration of Zn^2+^ that induced 50% reduction in viability in Zn^2+^-treated cells [101, 102].

Coating of zinc oxide nanoparticles with mercaptopropyl trimethoxysilane or SiO_2_ reduces their cytotoxicity [103]. On the contrary, Gilbert et al. [104] showed that in BEAS-2B cells, uptake of zinc oxide nanoparticles is the main mechanism of zinc accumulation. Also, they have suggested that zinc oxide nanoparticles dissolve completely generating Zn^2+^ ions which are bonded to biomolecules of the target cells. However, the toxicity of zinc oxide nanoparticles depends on the uptake and their subsequent interaction with target cells.

## Interaction Mechanism of Zinc Oxide Nanoparticles

**Fig. 2:**
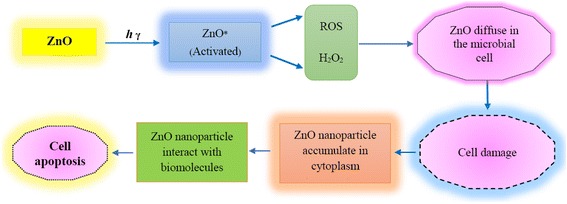
Mechanisms of zinc oxide nanoparticle antimicrobial activity

Nanoparticles may be toxic to some microorganisms, but they may be essential nutrients to some of them [55, 105]. Nanotoxicity is essentially related to the microbial cell membrane damage leading to the entry of nanoparticles into the cytoplasm and their accumulation [55]. The impact of nanoparticles on the growth of bacteria and viruses largely depends on particle size, shape, concentration, agglomeration, colloidal formulation, and pH of the media [106–108]. The mechanism of antimicrobial activity of zinc oxide nanoparticles has been depicted in Fig. 2.

**Fig. 3:**
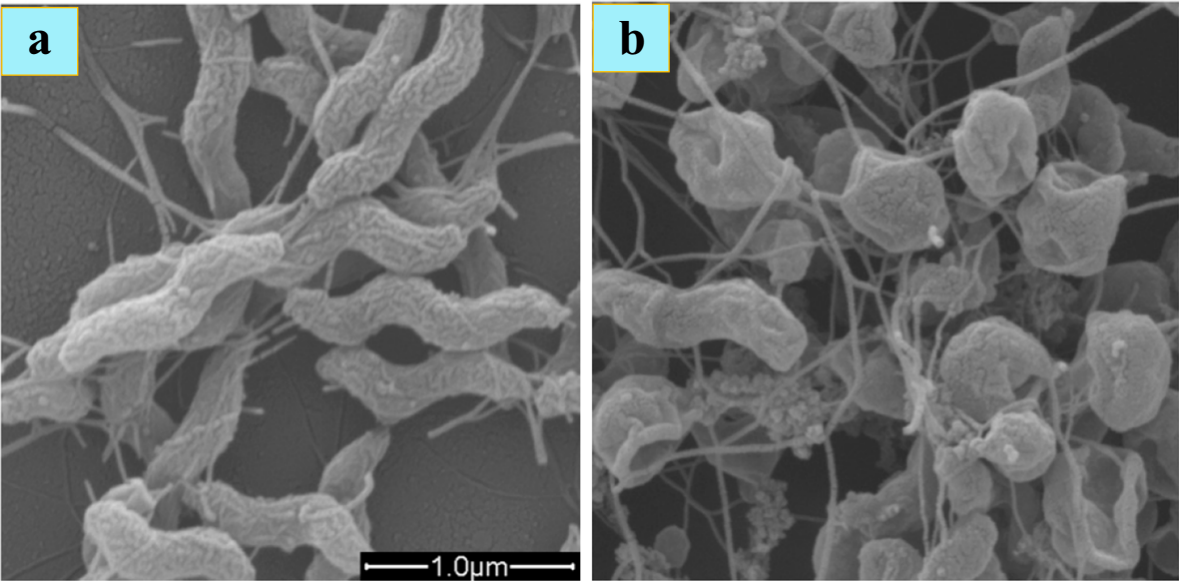
SEM images of *Campylobacter jejuni*. **a** Untreated cells from the same growth conditions were used as a control. **b** *C. jejuni* cells in the mid-log phase of growth were treated with 0.5 mg/ml of zinc oxide nanoparticles for 12 h under microaerobic conditions [114]

Zinc oxide nanoparticles are generally less toxic than silver nanoparticles in a broad range of concentrations (20 to 100 mg/l) with average particle size of 480 nm [55, 62, 63]. Metal oxide nanoparticles damage the cell membrane and DNA [63, 109–111] of microbes via diffusion. However, the production of ROS through photocatalysis causing bacterial cell death cannot be ignored [112]. UV-Vis spectrum of zinc oxide nanoparticle suspension in aqueous medium exhibits peaks between 370 and 385 nm [113]. It has been shown that it produces ROS (hydroxyl radicals, superoxides, and hydrogen peroxide) in the presence of moisture which ostensibly react with bacterial cell material such as protein, lipids, and DNA, eventually causing apoptosis. Xie et al. [114] have examined the influence of zinc oxide nanoparticles on *Campylobacter jejuni* cell morphology using SEM images (Fig. 3). After a 12-h treatment (0.5 mg/ml), *C. jejuni* was found to be extremely sensitive and cells transformed from spiral shape to coccoid forms. SEM studies showed the ascendency of coccoid forms in the treated cells and display the formation of irregular cell surfaces and cell wall blebs (Fig. 3a). Moreover, these coccoid cells remained intact and possessed sheathed polar flagella. However, SEM image of the untreated cells clearly showed spiral shapes (Fig. 3b). In general, it has been demonstrated from SEM and TEM images of bacterial cells treated with zinc oxide nanoparticles that they get ruptured and, in many cases, the nanoparticles damage the cell wall forcing their entry into it [114, 115].

**Fig. 4:**
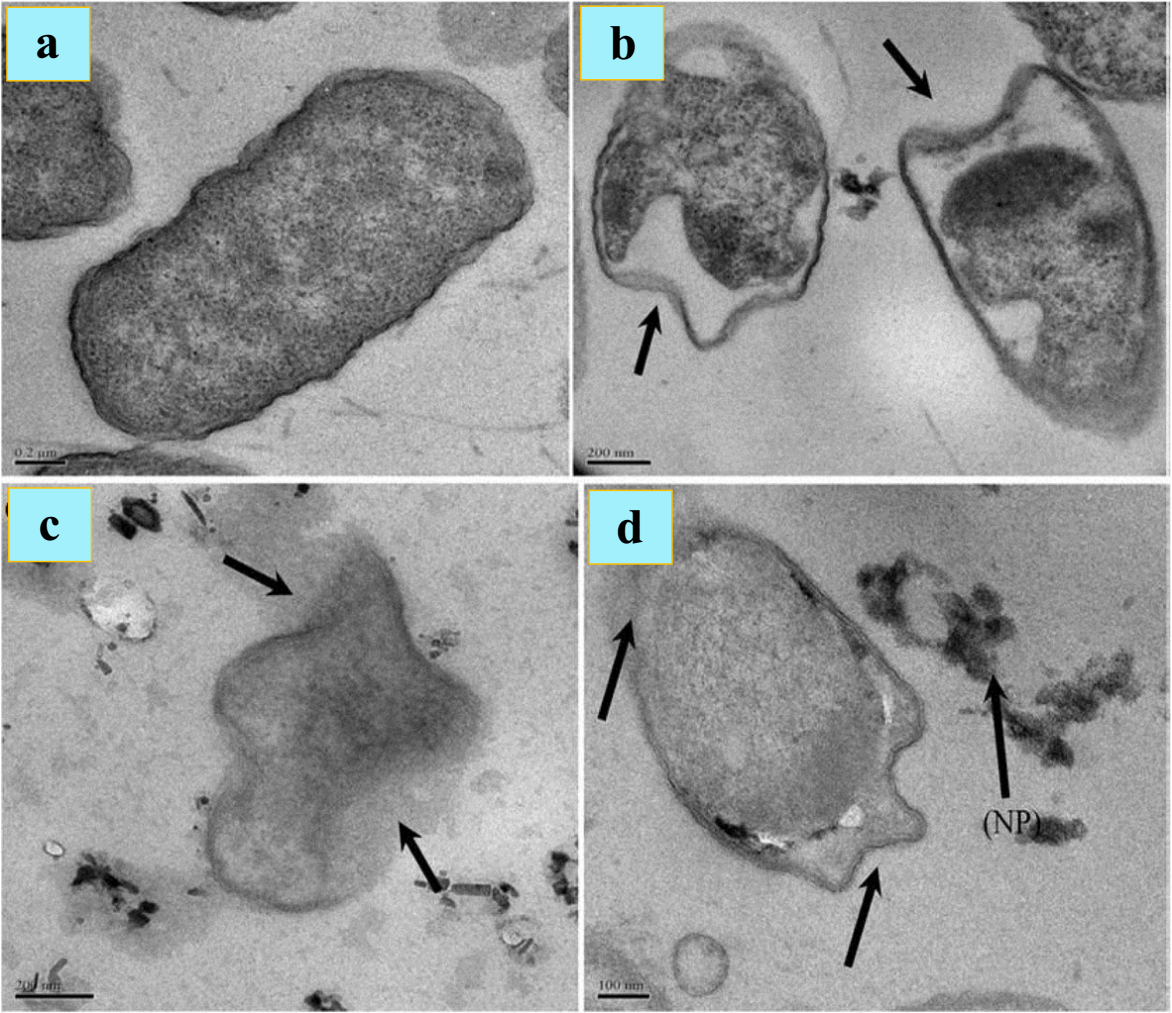
**a** TEM images of untreated normal *Salmonella typhimurium* cells. **b** Effects of nanoparticles on the cells (marked with arrows). **c**, **d** Micrograph of deteriorated and ruptured *S. typhimurium* cells treated with zinc oxide nanoparticles [115]

Zinc oxide nanoparticles have high impact on the cell surface and may be activated when exposed to UV-Vis light to generate ROS (H_2_O_2_) which permeate into the cell body while the negatively charged ROS species such as O_2_^2−^ remain on the cell surface and affect their integrity [116, 117]. Anti-bacterial activity of zinc oxide nanoparticles against many other bacteria has also been reported [1, 5, 114, 115]. It has been shown from TEM images that the nanoparticles have high impact on the cell surface (Fig. 4).

Sinha et al. [118] have also shown the influence of zinc oxide nanoparticles and silver nanoparticles on the growth, membrane structure, and their accumulation in cytoplasm of (a) mesophiles: Enterobacter sp. (gram negative) and *B. subtilis* (gram positive) and (b) halophiles: halophilic bacterium sp. (gram positive) and Marinobacter sp. (gram negative). Nanotoxicity of zinc oxide nanoparticles against halophilic gram-negative Marinobacter species and gram-positive halophilic bacterial species showed 80% growth inhibition. It was demonstrated that zinc oxide nanoparticles below 5 mM concentration are ineffective against bacteria. The bulk zinc oxide also did not affect the growth rate and viable counts, although they showed substantial decrease in these parameters. Enterobacter species showed dramatic alterations in cell morphology and reduction in size when treated with zinc oxide.

**Fig. 5:**
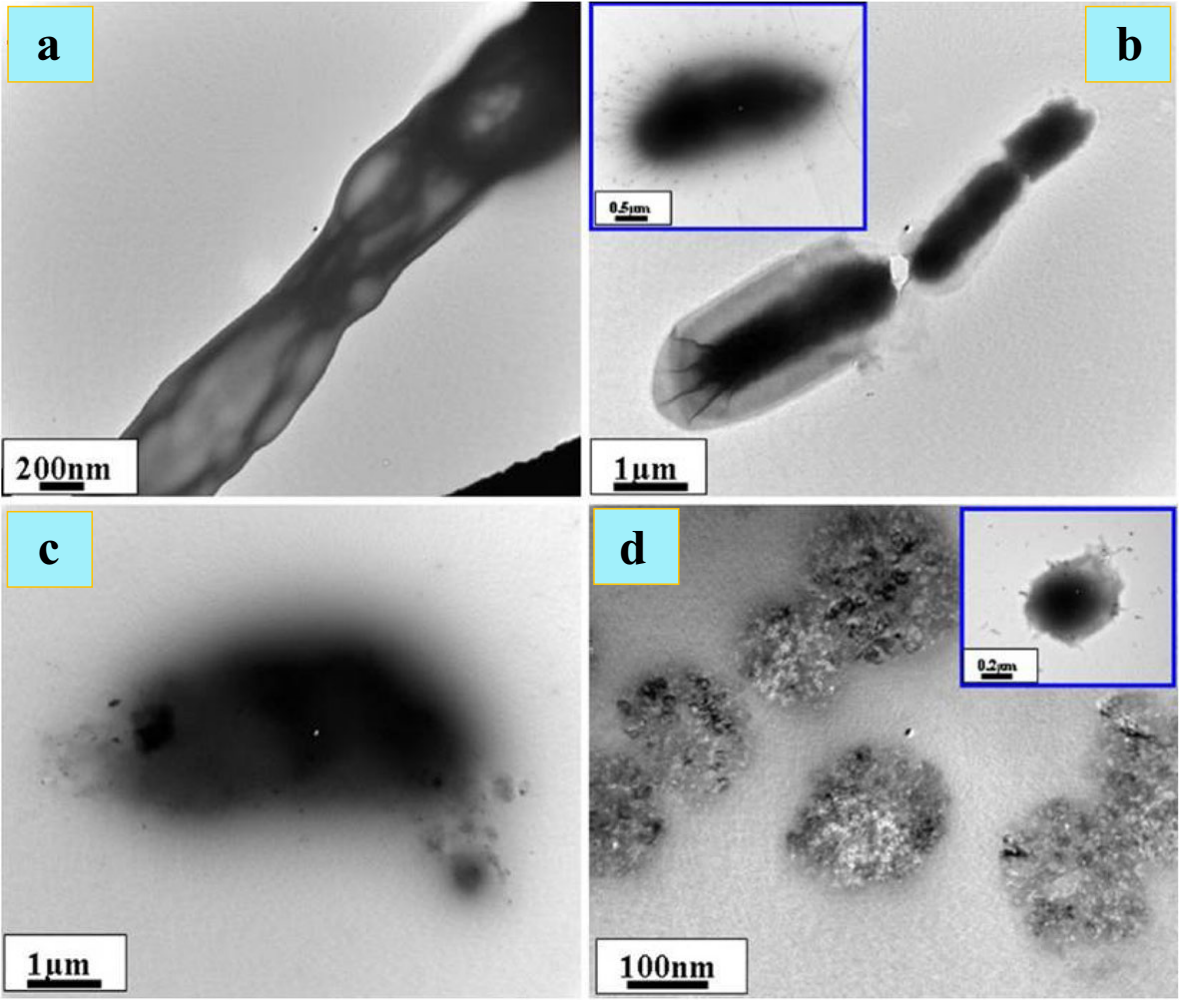
TEM images of *Escherichia coli* (**a**), zinc oxide nanoparticles with *E. coli* at different stages (**b** and inset), *Klebsiella pneumoniae* (**c**), and zinc oxide nanoparticles with *K. pneumoniae* (**d** and inset) [120]

TEM images shown by Akbar and Anal [115] revealed the disrupted cell membrane and accumulation of zinc oxide nanoparticles in the cytoplasm (Fig. 4) which was further confirmed by FTIR, XRD, and SEM. It has been suggested that Zn^2+^ ions are attached to the biomolecules in the bacterial cell via electrostatic forces. They are actually coordinated with the protein molecules through the lone pair of electrons on the nitrogen atom of protein part. Although there is significant impact of zinc oxide nanoparticles on both the aquatic and terrestrial microorganisms and human system, it is yet to be established whether it is due to nanoparticles alone or is a combined effect of the zinc oxide nanoparticles and Zn^2+^ ions [55, 106, 109, 119]. Antibacterial influence of metal oxide nanoparticles includes its diffusion into the bacterial cell, followed by release of metal ions and DNA damage leading to cell death [63, 109–111]. The generation of ROS through photocatalysis is also a reason of antibacterial activity [62, 112]. Wahab et al. [120] have shown that when zinc oxide nanoparticles are ingested, their surface area is increased followed by increased absorption and interaction with both the pathogens and the enzymes. Zinc oxide nanoparticles can therefore be used in preventing the biological system from infections. It is clear from TEM images (Fig. 5a, b) of *E. coli* incubated for 18 h with MIC of zinc oxide nanoparticles that they had adhered to the bacterial cell wall. The outer cell membrane was ruptured leading to cell lysis. In some cases, the cell cleavage of the microbes has not been noticed, but the zinc oxide nanoparticles can yet be seen entering the inner cell wall (Fig. 5c, d). As a consequence of it, the intracellular material leaks out leading to cell death, regardless of the thickness of bacterial cell wall.

Mechanism of interaction of zinc oxide nanoparticles with bacterial cells has been outlined below [120]. Zinc oxide absorbs UV-Vis light from the sun and splits the elements of water.

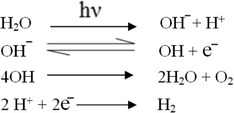



Dissolved oxygen molecules are transformed into superoxide, O_2_^−^, which in turn reacts with H^+^ to generate HO_2_ radical and after collision with electrons produces hydrogen peroxide anion, HO_2_^−^. They subsequently react with H^+^ ions to produce H_2_O_2_.

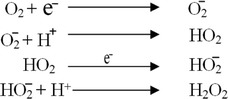



It has been suggested that negatively charged hydroxyl radicals and superoxide ions cannot penetrate into the cell membrane. The free radicals are so reactive that they cannot stay in free and, therefore, they can either form a molecule or react with a counter ion to give another molecule. However, it is true that zinc oxide can absorb sun light and help in cleaving water molecules which may combine in many ways to give oxygen. Mechanism of oxygen production in the presence of zinc oxide nanoparticles still needs experimental evidence.

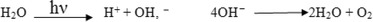



Zinc oxide at a dose of 5 μg/ml has been found to be highly effective for all the microorganisms which can be taken as minimum inhibitory dose.

## Conclusions

Zinc is an indispensable inorganic element universally used in medicine, biology, and industry. Its daily intake in an adult is 8–15 mg/day, of which approximately 5–6 mg/day is lost through urine and sweat. Also, it is an essential constituent of bones, teeth, enzymes, and many functional proteins. Zinc metal is an essential trace element for man, animal, plant, and bacterial growth while zinc oxide nanoparticles are toxic to many fungi, viruses, and bacteria. People with inherent genetic deficiency of soluble zinc-binding protein suffer from acrodermatitis enteropathica, a genetic disease indicated by python like rough and scaly skin. Although conflicting reports have been received about nanoparticles due to their inadvertent use and disposal, some metal oxide nanoparticles are useful to men, animals, and plants. The essential nutrients become harmful when they are taken in excess. Mutagenic potential of zinc oxide has not been thoroughly studied in bacteria even though DNA-damaging potential has been reported. It is true that zinc oxide nanoparticles are activated by absorption of UV light without disturbing the other rays. If zinc oxide nanoparticles produce ROS, they can damage the skin and cannot be used as sun screen. Antibacterial activity may be catalyzed by sunlight, but hopefully, it can prevent the formation of ROS. Zinc oxide nanoparticles and zinc nanoparticles coated with soluble polymeric material may be used for treating wounds, ulcers, and many microbial infections besides being used as drug carrier in cancer therapy. It has great potential as a safe antibacterial drug which may replace antibiotics in future. Application of zinc oxide nanoparticles in different areas of science, medicine, and technology suggests that it is an indispensable substance which is equally important to man and animals. However, longtime exposure with higher concentration may be harmful to living system.
